# Activation of Interferon Regulatory Factor 5 by Site Specific Phosphorylation

**DOI:** 10.1371/journal.pone.0033098

**Published:** 2012-03-08

**Authors:** Hui-Chen Chang Foreman, Sarah Van Scoy, Tsu-Fan Cheng, Nancy C. Reich

**Affiliations:** Department of Molecular Genetics and Microbiology, Stony Brook University, Stony Brook, New York, United States of America; McMaster University, Canada

## Abstract

The cellular defense to infection depends on accurate activation of transcription factors and expression of select innate immunity genes. Interferon regulatory factor 5 (IRF5), a risk factor for systemic lupus erythematosus, is activated in response to pathogen recognition receptor engagement and downstream effector molecules. We find the nucleotide-binding oligomerization domain containing protein 2 (NOD2) receptor to be a significant activator of IRF5. Phosphorylation is key to the regulation of IRF5, but the precise phosphorylation sites in IRF5 remained to be identified. We used mass spectrometry to identify for the first time specific residues that are phosphorylated in response to TANK-binding kinase-1 (TBK-1), tumor necrosis factor receptor-associated factor 6 (TRAF6), or receptor interacting protein 2 (RIP2). RIP2, a kinase known to function downstream of NOD2, was the most effective activator of IRF5-regulated gene expression. To determine if the phosphorylated residues are required or sufficient for IRF5 activity, aspartic acid phosphomimetic substitutions or inactivating alanine substitutions were tested. Phosphorylation of carboxyl serines 451 and 462 appear the primary trigger of IRF5 function in nuclear accumulation, transcription, and apoptosis. Results indicate polyubiquitination of IRF5 does not play a major role in its transcriptional activity, and that ubiquitination and phosphorylation are independent modifications.

## Introduction

Interferon regulatory factor 5 (IRF5) is an autoimmune susceptibility factor associated with increased risk of human systemic lupus erythematosus (SLE) [1], [2], [3], [4], [5]. Several animal disease models have demonstrated the role of IRF5 in autoimmunity development. Mice that spontaneously develop SLE either due to an underlying defect in Fas (MRL/lpr) or in the FcγRIIB receptor are protected in the genetic background of IRF5 deficiency [6], [7]. IRF5 deficient animals have defects in B cell differentiation and immunoglobulin isotype switching, which may highlight a role of IRF5 in autoantibody production characteristic of SLE [8], [9]. In addition, animals with a genetic knockout of IRF5 are protected from lethal shock induced by Toll-like receptor (TLR) ligands such as nucleic acids or lipopolysaccharide [10]. IRF5 is required for TLR signal transduction to induce proinflammatory cytokines including tumor necrosis factor-α (TNF-α), interleukin-6 (IL-6), and interleukin-12 (IL-12). Multiple aspects of IRF5 function may impact the complex development of SLE.

IRF5 is a latent transcription factor with constitutive expression in lymphocytes, macrophages and dendritic cells [11]. The IRF5 promoter possesses an interferon (IFN) stimulated response element and a p53 binding site, and has been shown to be induced in a variety of cell types [11], [12], [13]. IRF5 is activated from its latent state by post-translational modifications that include phosphorylation and ubiquitination [14], [15], [16], [17]. Activation of IRF5 in response to viral infection has been controversial [14], [15], [18]. Our studies indicate that viral infection with Newcastle Disease Virus (NDV) does not activate IRF5, although expression of kinases that function during viral infection, TBK-1 or NF-κB kinase-ε (IKKε), can phosphorylate IRF5 [14]. IRF5 has also been reported to be activated following TLR signaling and engagement of the TNF receptor associated factor 6 (TRAF6) adapter [10], [16]. TRAF6 is an E3 ubiquitin ligase that stimulates K63-linked ubiquitination and subsequent activation of numerous signaling molecules and protein kinases, including the receptor-interacting protein kinase (RIP2) [19], [20], [21], [22]. The RIP2 kinase is known to be a critical mediator of the innate immune response to nucleotide oligomerization domain (NOD)-like receptors and to require ubiquitination [21], [23], [24], [25], [26], [27]. In summary, TBK-1, TRAF6, and RIP2 have been implicated in the activation of latent IRF5.

Although phosphorylation appears to be key for IRF5 activation, the specific phosphorylated amino acids that lead to IRF5 activation remained to be determined. Mass spectrometry was not used prior to this study to directly identify IRF5 phosphorylation sites. Indications that serine clusters at the carboxyl terminus were important for activity were suggested based on homology with IRF3 and targeted mutations [14], [28]. In this study, we have identified specific amino acids by mass spectrometry that are phosphorylated in response expression of TBK-1, TRAF6, and RIP2. In addition we have evaluated the functional impact of site-specific mutations of these IRF5 phosphorylated residues. Substitution with alanine to create loss-of-function mutations or with aspartic acid to create gain-of-function mutations were assessed for the ability of IRF5 to induce gene expression, accumulate in the nucleus, or promote cell death [14], [29]. We further investigated the interplay between phosphorylation and ubiquitination, and the results indicate that phosphorylation and ubiquitination are independent functional modifications. Deciphering the molecular modifications that lead to IRF5 activation is expected to provide insight into its diverse functional roles and open the door for strategic drug design.

## Results

### Comparison of TBK-1, TRAF6, and RIP2 activation of IRF5

**Figure 1:**
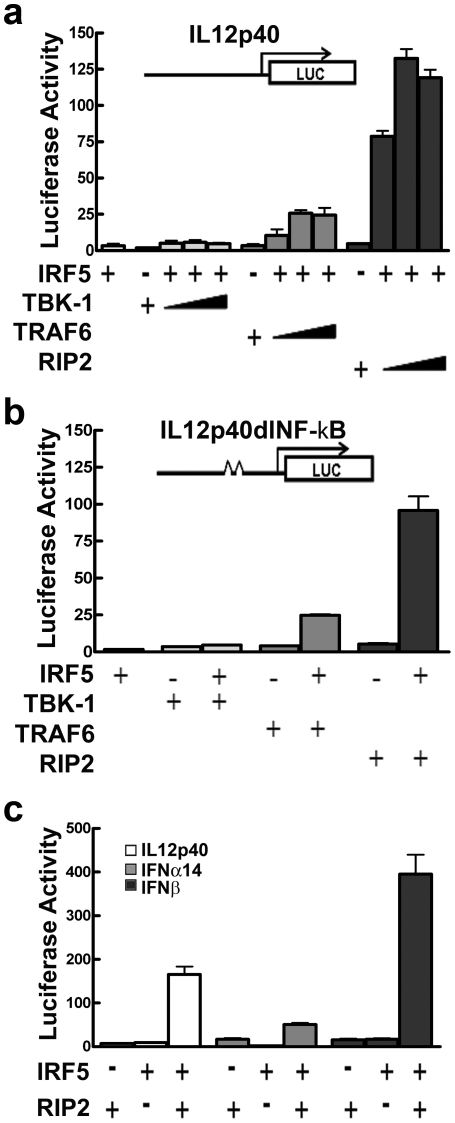
IRF5 comparative activation by TBK-1, TRAF6 or RIP2. **a**) IRF5 was co-expressed with vector or TBK-1, TRAF6, or RIP2 in HEK293 cells as indicated, and induction of the IL12p40 luciferase reporter was measured relative to *Renilla* gene. Response of IRF5 to activators was evaluated with increasing transfection ratio of activator to IRF5: 2-fold, 3-fold, and 4-fold. Values are means of duplicate determinations in three independent experiments. Protein expression controls are provided in Figure S1. **b**) Response of IL12p40 reporter gene lacking the promoter NF-κB sites (IL12p40dlNF-κB). Induction of IL12p40dlNF-κB luciferase with 4-fold transfection ratio activator to IRF5. Luciferase activity was measured as in (a). **c**) Activation of IRF5 by RIP2 leads to induction of reporter genes under the control of IL12p40, IFNα14 and IFNβ promoters. Cells were transfected with or without IRF5 and RIP2 as indicated, and induction was measured as in (a).

The response of cells to various ligands of pattern recognition receptors can be complex, involving cross-talk of diverse downstream signals. Thus in order to evaluate the impact of specific molecules on IRF5 transcriptional activity, we examined the effect of TBK-1, TRAF6, or RIP2 co-expression with IRF5 on a responsive reporter gene. IRF5 is required for induction of a number of cytokines including interleukin-12, and for this reason we used a luciferase gene reporter assay regulated by the promoter of the interleukin-12 (IL-12) p40 subunit gene [10]. The transfection results with the IRF5 activators are shown in Figure 1a. Although there was some stimulation by all of the activators, RIP2 kinase was clearly the most potent. Protein expression levels of IRF5 were equivalent (Figure S1). Since the IL12p40 promoter possesses both IRF5 and NF-κB binding sites, we tested the response of the promoter in which the two NF-κB binding sites were deleted. The IL12p40dlNF-κB reporter responded to IRF5 with a similar activation profile as the IL12p40 reporter gene, affirming the transcriptional function of IRF5 in this assay (Figure 1b).

The IL-12 p40 gene is a bona fide target of IRF5, but there is also evidence that IRF5 plays a role in the induction of a subset of type I interferon (IFN) genes [10], [17], [30]. To evaluate the contribution of IRF5 to the induction of IFN genes, we tested two other reporter genes regulated by either the human IFNα14 or IFNβpromoter. Both IFN reporters responded to IRF5 activated by RIP2 kinase, demonstrating IRF5 involvement in the induction of diverse cytokines (Figure 1c).

### TBK-1 modification of IRF5

**Figure 2:**
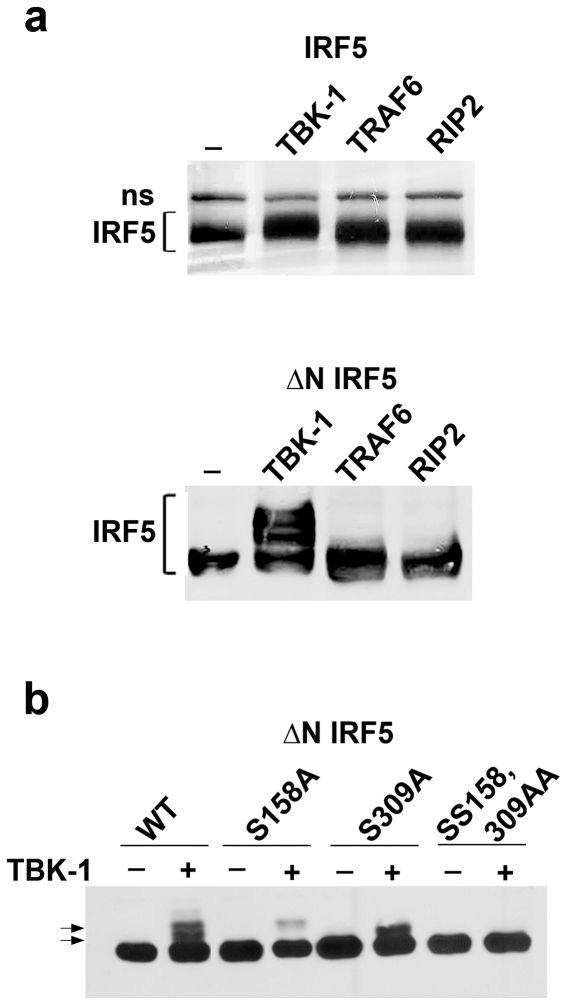
Phosphorylation of IRF5 by TBK-1. **a**) top) Mobility of IRF5 in SDS-PAGE with expression of control vector (-), TBK-1, TRAF6, or RIP2 expressed in HEK293 cells. T7-IRF5 was immunoprecipitated from cells, separated in SDS-PAGE and visualized by silver staining. Non-specific (ns) band is indicated. Bottom) Western blot of IRF5 from cells expressing amino-terminal deletion mutant of T7-IRF5 (121–514a.a.)(ΔN IRF5) with vector, TBK-1, TRAF6 or RIP2. Multiple slower mobility forms of IRF5 are indicated in presence of TBK-1. **b**) TBK-1 phosphorylation of IRF5. Site directed mutagenesis of IRF5 was performed to introduce the single or double mutations in S158A and S309A. Effects of these mutations on mobility of T7-ΔN IRF5 in comparison to wt is shown by Western blot with T7 antibody.

Phosphorylation of IRF5 appears to activate its ability to induce transcription of target genes. Since phosphorylation can change the mobility of proteins during gel electrophoresis, we evaluated the effect of activators on the migration of IRF5 through SDS-PAGE. IRF5 was immunoprecipitated from cells co-expressing TBK-1, TRAF6, or RIP2, separated by gel electrophoresis, and detected by silver staining (Figure 2a). There was an apparent decrease in the mobility of IRF5 only with expression of TBK-1. To more easily examine potential modified forms of IRF5 by gel electrophoresis we evaluated a smaller IRF5 protein lacking a.a. 1–120 (ΔN) (Figure 2b). Multiple slower migrating forms of ΔN IRF5 were readily apparent only with expression of TBK-1.

To identify the IRF5 residues modified in response to TBK-1, we used mass spectrometry. Two approaches were used as described in Methods; *in vitro* phosphorylation of bacterially expressed IRF5 by immunocomplexes containing TBK-1, and *in vivo* phosphorylation of IRF5 by co-expression with TBK-1 in tissue culture cells. Modified forms of IRF5 were separated by SDS-PAGE and submitted for analysis. Two phosphoserines were identified, S158 and S309 (Figure S2). To determine the contribution of these modified serines to the slow migrating forms of IRF5, we analyzed the behavior of alanine substitutions (Figure 2b). Each individual mutation, S158A or S309A, resulted in the loss of a distinct modified form and also eliminated the slowest migrating form, suggesting the slowest form is a result of dual phosphorylation sites. A double mutation SS158,309AA eliminated all apparent slow migrating forms of IRF5. Since TRAF6 and RIP2 do not generate the distinct slow migrating forms of IRF5 with phosphoserine 158 or phosphoserine 309, it appears that S158 and S309 are specific phosphorylation sites of TBK-1, not TRAF6 or RIP2.

### IRF5 phosphorylation sites and their role in transcriptional activity

**Figure 3:**
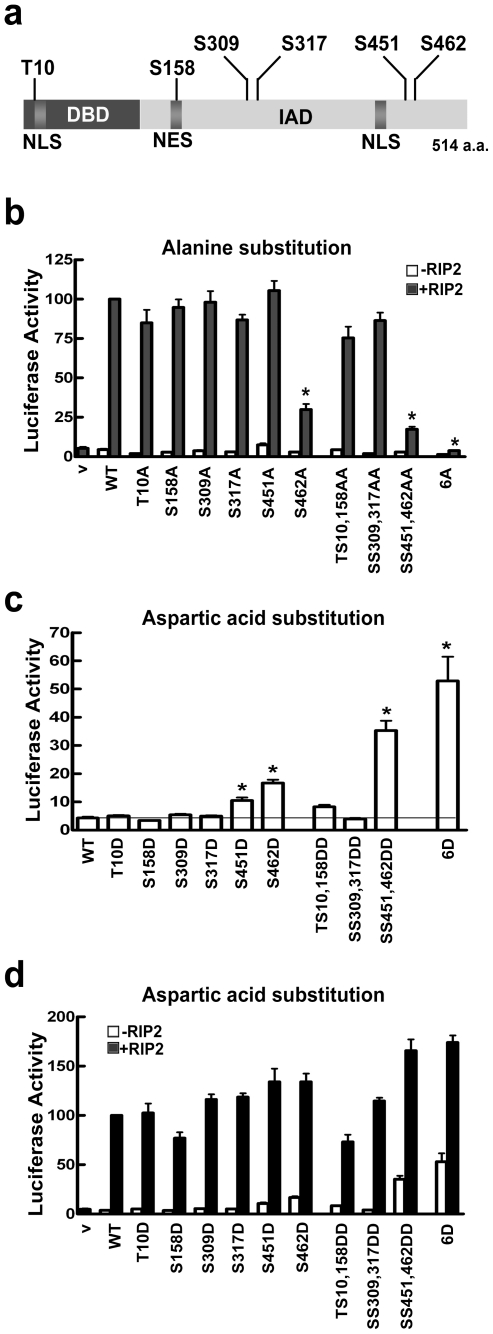
Effects of phosphorylation site mutations in IRF5. **a**) Linear diagram of IRF5 indicating the DNA binding domain (DBD), the interactive domain (IAD), the nuclear localization signals (NLS), and the nuclear export signal (NES). The amino acid phosphorylation sites of IRF5 identified by mass spectrometry are noted (T10, S158, S309, S317, S451, and S462) (Figure S2). **b**) The IL12p40 luciferase reporter assay was used to evaluate the effect of loss-of-function alanine substitutions in IRF5 in HEK293 cells. Single mutations or double mutations were introduced in IRF5 and their effect on the IL12p40 luciferase reporter was measured alone (white bar) or with RIP2 (dark bar). Luciferase activity is expressed relative to *Renilla* control and values are means of three to six independent experiments. *P<0.0001 **c**) Gain-of-function mutations were evaluated with aspartic acid substitutions for the phosphorylated amino acids. Effect of aspartic acid mutations on basal activity of IRF5 was measured with the IL12p40 luciferase reporter assay as in (b). *P<0.0001 **d**) Effect of IRF5 aspartic acid activating mutations in the absence or presence of RIP2 with the IL12p40 luciferase reporter assay as in (b).

Although TBK-1 phosphorylation of IRF5 altered its mobility in SDS-PAGE, the transcriptional activation of IRF5 was more robust in response to TRAF6 and RIP2. This suggested that distinct amino acids were phosphorylated in response to TRAF6 and RIP2 that did not alter IRF5 electrophoretic mobility, but significantly stimulated IRF5 transcriptional potential. To identify the phosphorylated amino acids that contribute to IRF5 activation, cells were transfected with IRF5 and either TBK-1, TRAF6, or RIP2. IRF5 was immunoprecipitated from each transfected cell lysate, combined, separated by SDS-PAGE, excised, and analyzed by mass spectroscopy. Four additional phosphorylation sites were identified, two located in a carboxyl terminal serine cluster (Figure 3a, Figure S2). In summary, we identified six phosphorylation sites in IRF5; Thr-10 (T10), Ser-158 (S158), Ser-309 (S309), Ser-317 (S317), Ser-451 (S451), and Ser-462 (S462). To address the contribution of these phosphorylation sites to IRF5 function, site-directed mutagenesis was performed to replace each modified amino acid with either alanine or aspartic acid. Alanine substitution was expected to eliminate effects of phosphorylation, and aspartic acid substitution could function as a phospho-mimetic with constitutive activity.

The effects of alanine loss of function substitutions for serine/threonine in IRF5 were evaluated with the IL12p40 reporter system and RIP2 (Figure 3b). The most significant effect was found with the S462A substitution. This single mutation reduced transcriptional induction by approximately 70% compared with wt IRF5. The double mutation SS451,462AA further reduced IRF5 transcriptional activity, and substitution of all six phosphorylated amino acids with alanine (6A) abrogated IRF5 function. The reduction in activity is not due to differences in protein expression (Figure S3a). The results indicate S462 as the most critical phosphorylation site, however other phosphorylation sites do contribute to IRF5 activity since the 6A mutant is dead in this assay.

In a converse approach, substitution with aspartic acid can serve as a phosphomimetic and reveal the positive contribution of the modified amino acids. Expression of the IRF5 aspartic acid substitutions in the absence of any activator was tested on induction of the IL12p40 promoter (Figure 3c). S462D was the most active single mutation, and combined with S451D, significantly induced the IL12p40 reporter gene. Notably the aspartic acid substitution of all six modified residues (6D) showed maximal activity indicating a modest contribution of other residues. RIP2 activation of the IRF5 aspartic acid substitutions showed a similar positive effect of S451D and S462D function in this assay (Figure 3d). Activity levels are not due to differences in protein expression (Figure S3b). Considered together, the substitution mutations indicate two carboxyl terminal serine residues S451 and S462, particularly S462, are critical for transcriptional activity of IRF5. The T10, S158, S309, S317 appear to play an auxiliary role.

**Figure 4:**
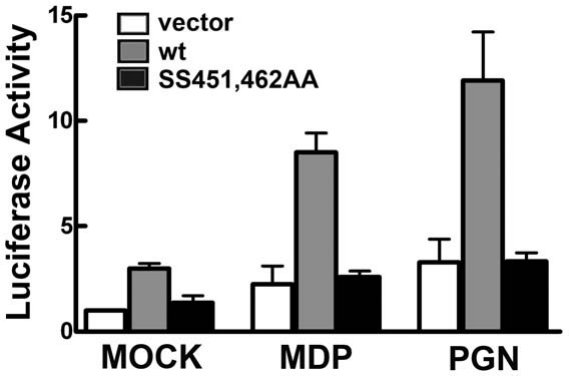
IRF5 activation by natural ligands of endogenous NOD2. Wt IRF5, alanine substitution SS451,462AA, or vector control were co-expressed with the IL12p40 luciferase reporter gene in the macrophage line RAW264.7. Cells were untreated or treated with muramyl dipeptide (MDP) or insoluble peptidoglycan (PGN) to stimulate endogenous NOD2. Luciferase activity was measured as in Figure 3. Values are means of three independent experiments; *P<0.0001.

To evaluate the biological significance of the critical phosphorylation sites in IRF5, we tested the response of IRF5 to activation by endogenous NOD2 pattern recognition receptors (Figure 4). NOD2 receptors are activated by muramyl dipeptide (MDP) which is a component of the peptidoglycan (PGN) cell wall of both Gram positive and Gram negative bacteria [31], [32], [33]. We tested NOD2 receptors since RIP2 is a critical downstream effector of NOD2 [23], [24], [25], [26], and RIP2 was found to be a potent stimulator of IRF5 transcriptional activity. Macrophage cells expressing the IL12p40 reporter with either wt IRF5 or the IRF5 alanine substitution SS451,462AA were treated with either of the NOD2 natural ligands, MDP or PGN. Results clearly show that in response to endogenous NOD2 signaling, IRF5 wt induced the transcriptional activity of the IL12p40 gene. However, the IRF5 mutant that lacks the serines 451 and 462 was not able to induce gene expression. The results demonstrate natural ligand activation of NOD2 stimulates IRF5 activity only if the serine residues 451/462 are available for phosphorylation, confirming the relevance of the phosphoserines 451 and 462 identified with mass spectrometry.

### Phosphorylation regulates IRF5 nuclear trafficking

In a static image latent IRF5 appears in the cytoplasm, however it dynamically shuttles in and out of the nucleus. This can be demonstrated by inhibiting IRF5 nuclear export with the antibiotic leptomycin B (LMB) [14], [15], [34]. Treatment of cells with LMB results in nuclear accumulation of latent IRF5, and this result indicates IRF5 has a constitutive nuclear localization signal (NLS) as well as a constitutive dominant nuclear export signal (NES). The location of two identified NLSs and one NES have been reported and are shown in Figure 3a [14], [15], [28]. To function as a transcription factor, nuclear transport is a critical event. Since the phosphorylated residue T10 is located near an NLS, and S158 is located within the NES, we evaluated the possible effect of phosphorylation on IRF5 nuclear trafficking by testing the phosphorylation site mutants.

**Figure 5:**
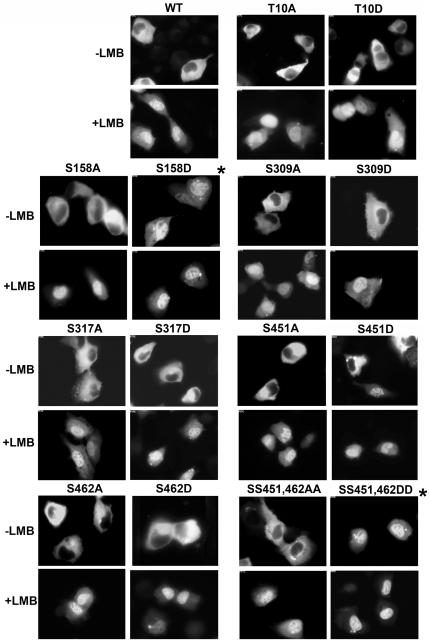
Cellular localization of wt IRF5 and phosphorylation mutants. Fluorescence imaging was used to evaluate cellular localization of wt GFP-IRF5 or point mutants in GFP-IRF5 expressed in HT1080 cells. Effects of amino acid substitutions with alanine (A) or aspartic acid (D) are shown in absence or presence of export inhibitor leptomycin B (LMB). *Asterisk notes significant gain of nuclear accumulation with S158D and SS451,462DD. Images represent random sampling of 12 to 15 fields and reflect more than 80% of the population in three independent experiments.

IRF5 wt and phosphorylation site mutants were tagged with GFP to enable direct visualization by fluorescence microscopy. Cellular localization was observed with or without LMB treatment to determine the impact of mutations on nuclear import. The effect of LMB on latent wt IRF5 can be seen to result in nuclear accumulation, indicating a functional constitutive NLS (Figure 5). Although T10A and T10D mutations are located near the amino terminal NLS of IRF5, they had no apparent effect on cellular localization. However, the S158D mutation in the region of the NES led to nuclear accumulation in the absence of LMB. Phosphorylation of S158 may therefore indirectly facilitate nuclear localization by inhibiting the NES function. However nuclear accumulation alone with S158D is not sufficient to induce the IL12p40 gene in the absence of activator (Figure 3c).

Mutations of the other phosphoserines identified by mass spectrometry were also evaluated for nuclear localization. The alanine or aspartic acid substitutions for S309, S317, and S462 had no notable effect on IRF5 localization, but S451D resulted in detectable nuclear IRF5 in 20–30% of the cells. The reason for the selective nuclear appearance of S451D in a subset of cells remains to be determined. The double mutant SS451,462DD that demonstrated significant transcriptional activation of the IL12p40 gene gained prominent nuclear accumulation independent of any activator.

### Relationship of IRF5 phosphorylation and ubiquitination

**Figure 6:**
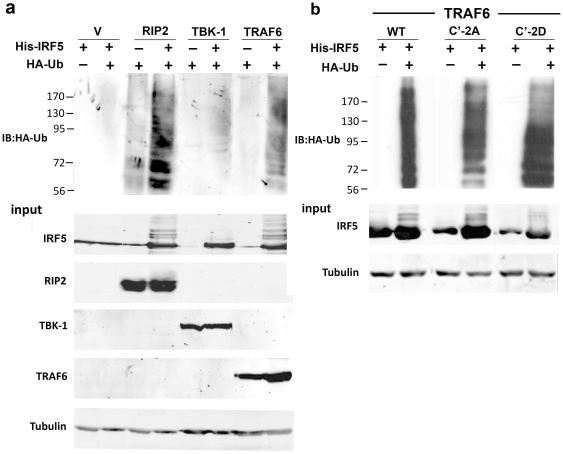
Ubiquitination of IRF5. **a**) Ubiquitination of wt IRF5 in response to RIP2, TBK-1, or TRAF6. His-tagged IRF5 or vector (v) and HA-tagged ubiquitin were expressed individually or co-expressed in HEK293 cells with myc-tagged RIP2, TBK-1, or TRAF6. IRF5 was captured from lysates by Ni^++^-NTA agarose beads and evaluated by Western/immunoblot blot (IB) for ubiquitination with anti-HA (IB:HA-Ub) or anti-IRF5 antibodies. Lysate input to Ni^++^-NTA beads is shown by Western blot with anti-IRF5, anti-RIP2, and anti-myc for TBK-1, TRAF6, and anti-tubulin. **b**) Polyubiquitination of wt IRF5, IRF5 SS451,462AA (C′-2A), or IRF5 SS451,462DD (C′-2D) with TRAF6 co-expression. His-tagged IRF5 constructs or vector co-expressed with HA-ubiquitin were collected on Ni^++^-NTA agarose beads as in (a). Polyubiquitination was detected by Western/immunoblot (IB) blot with anti-HA (IB:HA-Ub). Lysate input to Ni^++^-NTA beads is shown for IRF5 and tubulin.

IRF5 was reported to be polyubiquitinated via a K63 ubiquitin linkage in response to TLR signaling [16]. In that study IRF5 ubiquitination was reported to correlate with its transcriptional activity and nuclear accumulation. Our objective was to determine whether phosphorylation was required for ubiquitination or conversely whether ubiquitination was required for phosphorylation. To evaluate a possible interdependence, we first determined whether activation by RIP2 or TBK-1, stimulated the ubiquitination of IRF5 as did TRAF6. We co-expressed His-tagged IRF5 with these different activators and HA-tagged ubiquitin. IRF5 was isolated by affinity to Ni-NTA-agarose beads and evaluated by Western blot (Figure 6a). High molecular weight modified forms of IRF5 were obvious with co-expression of RIP2 and TRAF6, but were not significantly apparent with TBK-1. Western blot confirmed these modified forms of IRF5 to be polyubiquitinated.

It remained to be determined whether specific phosphorylation of IRF5 was required for its ubiquitination, or whether ubiquitination was sufficient for IRF5 activation. To evaluate these relationships we analyzed the IRF5 SS451,462AA (C′-2A) mutation that cannot be phosphorylated on the carboxyl serines, and the IRF5 SS451,462DD (C′-2D) mutation that is transcriptionally active (Figure 6b). TRAF6 was used as an activator of wt or mutant IRF5. The SS451,462AA mutant that is transcriptionally dead and cannot be phosphorylated at the critical serines was polyubiquitinated. Therefore although phosphorylation of these carboxyl serines is required for IRF5 transcriptional activity, phosphorylation of these residues is not required for ubiquitination. Polyubiquitin chains with different lysine linkages promote distinct effects; ubiquitin lysine 48 linkage targets substrates for degradation, whereas lysine 63 linkage promotes effects that include signaling and trafficking [35]. To determine the presence of K63-ubiquitination, IRF5 was co-expressed with HA-tagged ubiquitin that had all lysines substituted with arginine except lysine 63 (HA-K0R63K). Results showed both SS451,462AA (C′-2A) and SS451,462DD (C′-2D) were modified by K63 polyubiquitination (Figure S4). Together the results indicate phosphorylation is not required for ubiquitination, and ubiquitination itself is not sufficient for IRF5 transcriptional activity.

**Figure 7:**
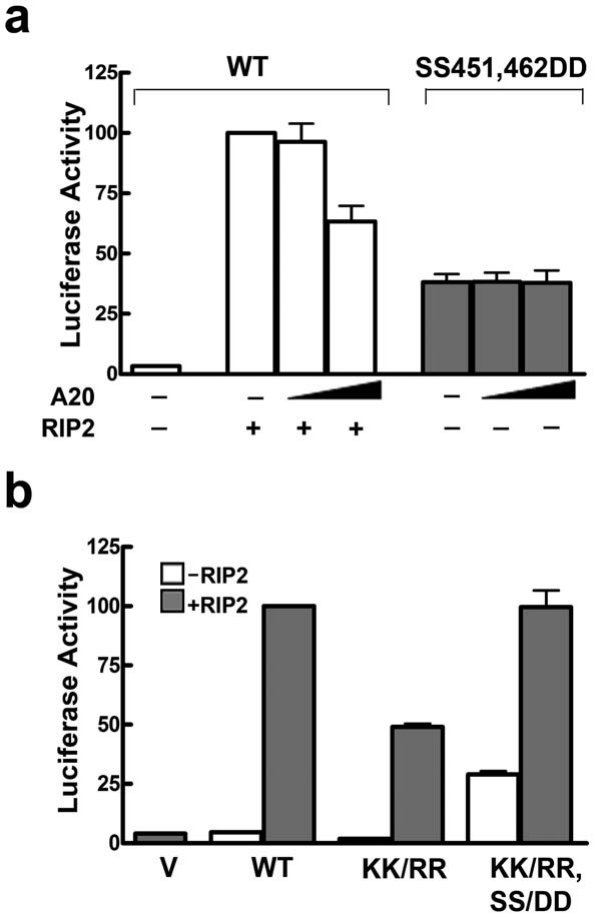
Activity of IRF5 SS451,462DD independent of ubiquitination. **a**) The activity of wt IRF5 or IRF5 SS451,462DD on the IL12p40 luciferase reporter assay was evaluated in the presence or absence of RIP2 with increasing expression of A20 (1-fold, and 1.33-fold relative to RIP2). Luciferase activity was measured relative to co-transfected *Renilla* gene. **b**) IRF5 activity independent of the characterized ubiquitination site at K410,K411 in IRF5 v4. KK410,411RR site mutation in IRF5 v4 is equivalent to KK427,428RR in IRF5 v5. Induction of the IL12p40 luciferase reporter gene by wt IRF5, IRF5 KK/RR, or IRF5 KK/RR, SS451,462DD (KK/RR, SS/DD) with or without expression of RIP2. The data is presented relative to *Renilla* control and vector alone (v). Values are means of duplicate determinations in two independent experiments.

To further investigate the interplay between phosphorylation and ubiquitination of IRF5, we tested the effect of the ubiquitin-editing enzyme A20, known to possesses K63-deubiquitinating activity [36], [37], [38]. RIP2 was used as an upstream activator of IRF5 and the effect of A20 co-expression was evaluated on the ability of wt IRF5 to induce the IL12p40 reporter gene (Figure 7a). A20 decreased the ability of RIP2 to stimulate IRF5 transcriptional activity. This result could be due to deubiquitination of IRF5 or RIP2 [21], [23], [27], [39]. If IRF5 requires ubiquitination for activity, the constitutively active SS451,462DD mutant would also be inhibited by A20. However, the active phosphomimetic mutant was resistant to A20 expression (Figure 7a). These results suggest continuous K63-ubiquitination of IRF5 is not required for IRF5 transcriptional activity.

Another line of evidence indicated that direct ubiquitination of IRF5 is not necessary for its transcriptional function. Specific lysine residues of IRF5 were previously reported to be required for K63-ubiquitination (designated K410, K411) [16]. To test the requirement of these lysines for transcriptional activity, we substituted the corresponding lysine residues with arginine to generate KK/RR. Although activity was reduced in comparison to wt IRF5, IRF5 KK/RR was able to significantly increase transcription of the reporter gene (Figure 7b). Indeed if the KK/RR mutation was combined with the activating SS451,462DD mutation, the double mutant had full transcriptional activity in the presence of RIP2 in comparison to wt IRF5. These data indicated that the lysine residues identified as ubiquitination target sites in IRF5 are not required for transcriptional activity. Results are not due to differences in protein expression (Figure S5). Together the data suggest that ubiquitination and phosphorylation are independent modifications, and the critical commitment for IRF5 nuclear accumulation and transcriptional activation is phosphorylation of the carboxyl serines 451 and 462.

### Phosphorylation affects IRF5-mediated apoptosis

**Figure 8:**
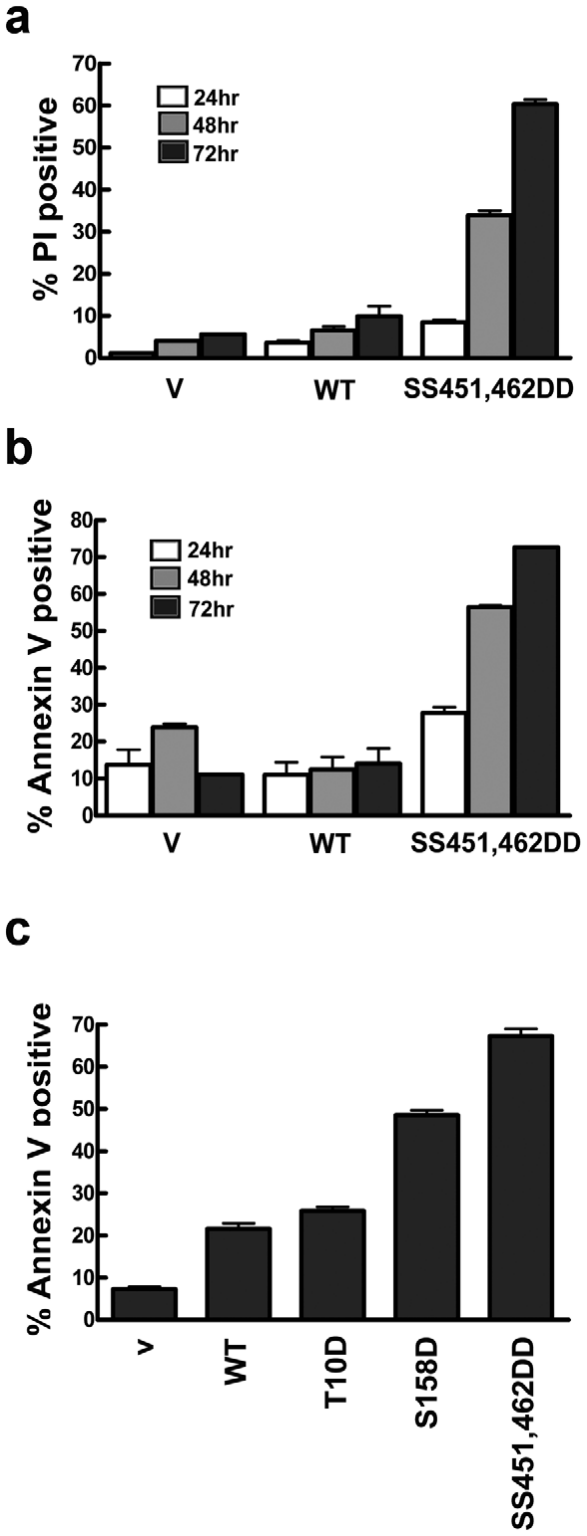
Pro-apoptotic effect of constitutively active IRF5 mutants. **a**) HeLa cells were transfected with plasmids encoding GFP vector (v), wt GFP-IRF5 (WT) or GFP-IRF5 double mutant SS451,462DD. Cells were stained with propidium iodide after one, two, or three days post-transfection and cells positive for GFP and PI were quantified by flow cytometry. **b**) Cells were transfected as in (a) and were stained with annexin V. Cells positive for GFP and annexin V were measured by flow cytometry. **c**) Three days following transfection with GFP vector, wt GFP-IRF5, or GFP-IRF5 with the noted activating aspartic acid substitutions cells were stained with annexin V. Cells positive for GFP and annexin V were quantified by flow cytometry. All results are means of triplicate determinations in three independent experiments.

Studies by our group and others have linked expression of IRF5 with another cellular function, the promotion of apoptosis [14], [29], [40], [41]. To determine if the specific phosphorylation of IRF5 contributes to its pro-apoptotic effects, we evaluated the IRF5 aspartic acid gain-of-function mutations. Cells expressing wt IRF5 or the IRF5 mutants tagged with GFP were analyzed with flow cytometry for cell death by permeability to propidium iodide (Figure 8a) or for apoptosis by staining with annexin V (Figure 8b) [42]. Expression of wt IRF5 had a modest effect on cell death, however the transcriptionally active SS451,462DD mutation had a dramatic pro-apoptotic effect. The promotion of apoptosis correlated with the transcriptional activity of SS451,462DD. Unexpectedly the S158D mutation that did not transcriptionally induce the IL12p40 gene, also promoted apoptosis, albeit to a lesser extent. The S158D mutation is within the NES of IRF5 and localize the protein to the nucleus presumably due to an inhibition of nuclear export (Figure 5). The results indicate both transcriptional activation and nuclear presence contribute to the pro-apoptotic effects of IRF5. The increases in apoptosis seen with these aspartic acid mutants were not due to differences in protein expression (Figure S6).

## Discussion

Engagement of pattern recognition receptors (PRRs) stimulates innate immune responses that promote clearance of microbial infections, but in addition these responses can contribute to inflammation and autoimmunity [43], [44]. IRF5 has been shown to respond to TLR and NOD signaling, and polymorphisms of IRF5 are associated with increased risk of autoimmune disease [1], [3], [4], [5], [9], [10], [17]. Since IRF5 may be a potential target of autoimmune intervention, knowledge of the mechanisms that activate IRF5 is needed [21], [30], [45]. Post-translation phosphorylation has been shown to regulate the molecular switch in IRF5 from latency to activation, however the precise phosphorylation sites in IRF5 remained to be determined. In this study we identified amino acids in IRF5 that are phosphorylated in response to signaling molecules downstream of PRRs. Our subsequent mutagenesis studies clearly establish the contribution of IRF5 modified amino acids to transcriptional induction, nuclear trafficking, and apoptosis.

We investigated the effects of several potential activators of IRF5; TBK-1, TRAF6, and RIP2. TBK-1 was previously identified as a kinase that phosphorylates the carboxyl terminus of the related transcription factor, IRF3, in response to viral infection [46], [47], [48], [49], [50]. TRAF6 is an E3-K63 ubiquitin ligase that can activate effector enzymes downstream of TLR and NOD signaling [19], [20], [51]. RIP2 kinase plays an essential role in the biological responses to NOD signaling [21], [23], [52]. We tested the effect of these activators on induction of the IL12 p40 subunit gene by IRF5. TBK-1 had a modest effect on the ability of IRF5 to induce the IL12p40 reporter, however TRAF6 and especially RIP2 were far more effective activators of IRF5 (Figure 1).

Although the gene induction by IRF5 in the presence of TBK-1 was modest, phosphorylation by TBK-1 produced a significant shift in the mobility of IRF5 in SDS-PAGE. For this reason we identified the residues that are phosphorylated on IRF5 by TBK-1 with mass spectrometry. Ser-158 and Ser-309 were found to be phosphorylated, and mutations of these serines eliminated specific shifts in IRF5 mobility by SDS-PAGE. These residues did not appear to be modified by TRAF6 or RIP2. In addition, these residues did not reside in the carboxyl terminus of IRF5, a region that was expected to contribute to its modification and activation [14], [53].

TRAF6 and especially RIP2 were far more effective activators of IRF5 than TBK-1 (Figure 1), and mass spectrometry identified additional IRF5 amino acids phosphorylated with TRAF6 and RIP2. In total, six phosphorylated residues were identified in IRF5 in response to co-expression of upstream activators TBK-1, TRAF6, and RIP2: Thr-10, Ser-158, Ser-309, Ser-317, Ser-451, and Ser-462 (Figure 3). To determine the contribution of each of these amino acids to the transcriptional function of IRF5, each was substituted with alanine to evaluate loss-of-function or with aspartic acid to evaluate a gain-of-function. Judging by the effect of the mutations, S462D stimulated considerable gene induction in the absence of activators while S462A had a severe negative effect on IRF5. There was also a cooperative effect of S462D with S451D, supporting the tenet that phosphorylation of carboxyl serine residues is a key element for IRF5 activity [14], [28], [53]. Although these two phosphoserines are the critical modifications, phosphorylation of other residues outside the carboxyl terminus plays an auxiliary role. This is demonstrated by the most significant effects with substitution of all of the modified residues as 6A or 6D. Although IRF5 6D stimulated significant gene expression, the addition of RIP2 boosted the response. This may be due to other transcription factors stimulated by RIP2 that can interact directly with IRF5 or augment IRF5 effects indirectly. The cellular response to pathogens via PRRs results in stimulation of complex signal pathways and activation of diverse adapters, ubiquitin ligases, and kinases. Pathogens can also activate different PRRs coordinately, promoting pathway cross-talk. The multiple phosphorylation sites on IRF5 may reflect an evolved response to activation of diverse PRRs and kinases.

As a transcription factor IRF5 must gain entrance to the nucleus, and nuclear transport is one mode of regulation. Although IRF5 has two reported NLSs and is transported to the nucleus continually, it also has a dominant NES [14], [15], [28]. Therefore the latent IRF5 protein continually shuttles in and out of the nucleus, but is prominent in the cytoplasm. We evaluated the effect of the substitution mutants on the cellular localization of IRF5 (Figure 5). The SS451,462DD double mutant showed dominant accumulation in the nucleus in the absence of activators. This was not unexpected since the double mutant is transcriptionally active. However, there were some unexpected findings. The S462D mutant did not accumulate in the nucleus although it induced gene expression independent of activators. It is possible that the continuous nuclear shuttling of S462D provides sufficient residence time in the nucleus to effect gene expression. The S451D activating mutant accumulated in the nucleus of only 20–30% of the expressing cells. The reason for the heterogeneity of localization in the culture remains to be determined. In addition, another phosphomimetic mutation was prominently nuclear, S158D. This was unexpected since this modification did not activate IRF5's transcriptional activity. However, serine 158 is located within the IRF5 NES (LQRMLPSLSLT), and so the result suggests that the serine phosphorylation impairs nuclear export of IRF5.

Since K-63 polyubiquitination was reported to be necessary for IRF5 activation, we investigated the relationship between IRF5 phosphorylation and ubiquitination [16]. TRAF6 and RIP2, potent activators of IRF5 transcriptional activity, were found to stimulate IRF5 ubiquitination whereas TBK-1 did not (Figure 6). To determine whether the phosphorylation of the activating carboxyl serines was necessary for ubiquitination, we evaluated TRAF6 ubiquitination of SS451,462AA in comparison with SS451,462DD or wt. Polyubiquitination of SS451,462AA was clearly detectable, demonstrating that phosphorylation of these serines is not required for ubiquitination.

We used two other approaches to evaluate ubiquitination and transcriptional activity of IRF5 (Figure 7). A20 is an ubiquitin-editing enzyme with K-63 deubiquitinase activity [54]. It is known to inhibit signaling from the PRRs [27], [55], [56]. When co-expressed with RIP2, it inhibited the ability of wt IRF5 to induce transcription. To determine if A20 impacted upstream signaling molecules like RIP2 or directly influenced IRF5, we tested the constitutively active mutant, SS451,462DD in the absence of RIP2. The A20 deubiquitinase did not reduce the transcriptional activity of SS451,462DD, suggesting ubiquitination is not necessary for IRF5 activity. A second approach was to evaluate the effect of lysine mutations in IRF5 reported to be targets of ubiquitination. The TRAF6 K63-ubiquitination site in IRF5v4 has been identified and characterized [16], [28]. We tested IRF5 activity following mutation of these corresponding lysines in IRF5v5 (KK427,K428RR) (KK/RR). Although the transcriptional activity of IRF5 KK/RR was 2-fold less than wt, IRF5 KK/RR was still activated by RIP2, and activity was complete in the context of the activating mutation (KK/RR, SS/DD). Together, the results indicate that carboxyl terminal phosphorylation of IRF5 and not ubiquitination is the critical modification that determines IRF5 transcriptional activity.

Since another property of IRF5 is its ability to promote apoptosis, we tested the effects of substitution mutants on this function (Figure 8). Expression of the transcriptionally active mutant SS451,462DD was found to stimulate an apoptotic cell death. This finding was not unexpected since the ability of IRF5 to regulate transcription may be linked to pro-apoptotic gene expression. However, another phosphomimetic mutation that significantly enhanced cell death was S158D. This was unexpected since S158D was not transcriptionally active in our assay. However S158D did accumulate in the nucleus, and therefore its ability to promote apoptosis may indicate that this modification allows IRF5 to interact with other nuclear factors that influence cell death. This mechanism of action remains to be determined.

Phosphorylation of IRF3, IRF5, and IRF7 triggers a conformational change that promotes dimerization and binding to CBP/p300 [48], [53], [57]. Several isoforms of IRF5 have been identified, most resulting from alternative splicing [14]. We have investigated variant 5, the longest form of IRF5, and amino acid numbering is relative to this isoform. The crystal structure of a fragment of IRF5 (a.a. 222–467) has been solved with variant 4 (v4) using a phosphomimetic substitution S430D, which corresponds to serine 456 in variant 5 (v5) [53]. Our mass spectrometry results did not identify phosphorylation of the serine 456v5, but phosphorylation of flanking serines, 451v5 and 462v5. Although the crystal structure of IRF5 was not solved with an authentic phosphorylation site, certain predictions can be made from their analyses. The structural data predicts phosphorylation of serine 451v5 contributes to destabilization of the autoinhibitory conformation of IRF5. Our results with a phosphomimetic of this serine (S415D) showed an increase in transcriptional activity and a modest increase in nuclear accumulation. The crystal structure predicts phosphorylation of serine 462v5 plays a significant role in stabilization of the formed IRF5 dimers [53]. The serine 462v5 is positioned within hydrogen bonding distance of arginine 354v5, an arginine that is conserved in human IRF3 and IRF7. Our results with the phosphomimetic S462D demonstrated a considerable increase in transcriptional activity. More significantly, a phosphomimetic substitution of both serine 451 and 462 together (SS451,462DD) provided a dramatic increase in nuclear accumulation, transcriptional activity, and pro-apoptotic effects. These data support the tenet that phosphorylation of serine 451 relieves the autoinhibitory conformation, and phosphorylation of serine 462 stabilizes the IRF5 dimers. Phosphorylation of these serines together serves as a trigger for conformational change and dimerization.

In this study our objective was to elucidate the molecular modifications that regulate IRF5 transition from latency to an active transcription factor. For the first time specific phosphorylation sites of IRF5 have been identified by mass spectrometry, and their contributions to gene induction and apoptosis have been evaluated. In addition, the effectiveness of RIP2 as an upstream activator of IRF5 suggests that IRF5 plays a preferential role in NOD-like receptor signaling. This knowledge advances our understanding of the molecular mechanisms that trigger IRF5 activity in health and disease.

## Materials and Methods

### Cell Culture and reagents

Human HEK293, HT1080, HeLa and murine RAW264.7 cells were obtained from ATCC. Cells were grown in Dulbecco's modified Eagle's medium with 8% fetal bovine serum, penicillin (100 U/ml) and streptomycin (100 mg/ml) (Invitrogen). To measure the effect of NOD2 signaling, 50 µg/ml of muramyl dipeptide (MDP) or 15 µg/ml insoluble peptidoglycan (InvivoGen) was added to RAW264.7 cultures. Leptomycin B (LMB) was used at 10 ng/ml (gift from B. Wolff-Winiski, Novartis Research Institute).

### Expression plasmids

Plasmids T7-His-tagged pcDNA3 vector, T7-His-tagged IRF5v.5, GFP-IRF5, and FLAG-TBK-1 have been described [14]. The T7-His-tagged ΔN IRF5 was generated by PCR (primers in Table S1). The ΔN IRF5 DNA fragment spanning from 201 to 514 amino acids of IRF5 was subcloned into the T7-His-pcDNA3 and verified by sequencing. IRF5 point mutants were constructed using primers (Table S1) by Quick Change mutagenesis kit (Stratagene) and verified by sequencing. His-tagged ΔNIRF5 was cloned into bacterial expression vector pET-15b (Novagen). Luciferase reporter genes were driven by the human IFNα14 promoter (−457 to +71) (Dr. Racine Brzostek, Stony Brook University) and IFNβ promoter [14]. The following plasmids were generous gifts: FLAG-TBK-1 (Dr. Michael Karin, University of California San Diego); c-myc-TBK-1 (Dr. Erich Mackow, Stony Brook University); c-myc-TRAF6 (Dr. John Reed, Burnham Institute); HA- or omni-tagged-RIP2 (Dr. Derek Abbott, Case Western Reserve University) [39]; IL12p40 and IL12p40dlNF-κB luciferase reporter genes (Dr. Keiko Ozato, NIH) [58]; HA-tagged ubiquitin (HA-Ub) and HA-tagged K0R63K (HA-K63Ub) (Dr. Dafna BarSagi, New York University) [59]; A20 (Dr. Anatoly Grishin, University of Southern California) [56].

### Transfection and Luciferase reporter assay

Transfection of RAW264.7 cells was performed by electroporation (Amaxa Cell Line Nucleofector Kit V, Lonza). HEK293, HT1080 and HeLa were transfected using *Trans*It-LT1 transfection reagent (Mirus; Madision, WI, USA). The luciferase activity was measured by a Lumat model LB 9507 luminometer using Dual-Luciferase Reporter Assay System (Promega). Results were normalized to co-transfected pRLTK reporter gene (*Renilla* luciferase, Promega; Madison, WI, USA). Values are means of three to six independent experiments, and bars show one standard error of the mean, and are expressed as the activity relative to pcDNA3 alone.

### Direct Fluorescence imaging

HT1080 cells on coverslips were transfected with GFP-IRF5 constructs and 24 hours later treated with leptomycin B for 1 hour. Cells were fixed in 3.7% formaldehyde/PBS and stained with 2 µg/ml of Hoechst 33342 at room temperature for 15 minutes. Coverslips were washed and mounted in Vectashield antifade solution (Vector Laboratories; Burlingame, CA). GFP-tagged proteins were observed with Zeiss Axiovert 200M and Axiovision Version 4.5 and images captured with Adobephotoshop.

### Apoptosis assay

HeLa cells were transfected with GFP-IRF5 constructs, washed with media six hours post-transfection, and cell death was measured 1, 2 or 3 days post-transfection by propidium iodide staining and evaluation with a FACSCalibur flow cytometer (BD Biosciences, Breda, The Netherlands) [42]. Apoptosis was evaluated by staining with allophycocyanin (APC)-conjugated annexin V (BD Pharmingen; San Diego, CA) and flow cytometry. The gate was set for GFP expression, and 10,000 cells in each population were analyzed with BD CellQuest software.

### Immunoprecipitation, Silver Staining and Western blot

Antibodies used included anti-IRF5 (ProteinTech Group Inc.; Chicago, IL), anti-T7 (Novagen), anti-RIP2 (sc-22763, Santa Cruz Biotechnology), anti-omni (sc-7270, Santa Cruz Biotechnology), anti-c-Myc (sc-40, Santa Cruz Biotechnology), anti-HA (12CAS, Roche), anti-FLAG (F3165, Sigma), and secondary anti-mouse (Rockland) and anti-rabbit (Invitrogen) antibodies for Western blot analysis with Odyssey Imager (Li-COR Biosciences). For immunoprecipitation, cells were lysed in 50 mM Tris (pH 7.5), 400 mM NaCl, 5 mM EDTA, 0.5% Nonidet P-40, 50 mM sodium fluoride, 10% glycerol, 10 mM β-glycerolphosphate, 1 mM sodium vanadate, 1 mM PMSF and protease inhibitor mixture (Sigma-Aldrich). Lysates were clarified by centrifugation at 12,000 g for 10 min prior to antibody addition. Immunocomplexes were collected with protein-G beads, eluted, and separated on 8.5% SDS-PAGE. Proteins were transferred to Immobilon-P (Millipore) for Western blotting and reactive signals were detected with the Odyssey Imager (Li-COR Biosciences) and analyzed using Image J software (NIH). Alternatively, secondary antibodies linked to HRP were used (Amersham/GE Healthcare) and the membrane was incubated in enhanced chemiluminescence reagents and exposed to film. Proteins visualized without Western blotting were detected by silver staining (SilverQuest, Invitrogen)

### Ubiquitination assays

HEK293 cells were co-transfected with plasmids encoding His-tagged IRF5 and c-myc-tagged TBK-1 or c-myc-tagged TRAF6 or HA-tagged RIP2 with wild type HA-tagged ubiquitin or HA-tagged K0R63K ubiquitin. 48 hours post-transfection cells were harvested and His-tagged IRF5 proteins were isolated on Ni-NTA agarose beads (Qiagen). Elutes from the Ni-NTA agarose beads were subjected to 8.5% SDS-PAGE analysis and Western blot.

### Mass Spectrometry Analysis

Analysis of IRF5 phosphorylated amino acids by TBK-1 was performed with two approaches. Bacterially expressed and purified His-tagged ΔNIRF5 was phosphorylated *in vitro* by FLAG-TBK-1 immunoprecipitated from mammalian HEK293 cells. Proteins were separated on 7.5% SDS-PAGE and detected by Coomassie brilliant blue staining. A slower mobility IRF5 protein band was provided to the University of Massachusetts Medical School Proteomics Lab for in gel protein cleavage with trypsin and analysis by LS/MS/MS. Their data interpretation indicated phosphorylation of serine 156 or 158 and we confirmed 158 was modified by mobility shift analyses (Figure S2). *In vivo* phosphorylation of T7-His-IRF5 S158A mutant was analyzed by co-expression with FLAG-TBK-1 in HeLa cells. T7 antibodies conjugated to agarose beads (Novagen) were used to collect T7-IRF5 immunocomplexes from cell lysates. IRF5 was visualized in SDS-PAGE by staining with SimplyBlue (Invitrogen), and slow mobility IRF5 protein band was eluted, treated with iodoacetamide, and submitted for analysis to ProtTech Inc. (Norristown, PA). The sample was digested with trypsin and chymotrypsin to generate peptides that were reconstituted in 2% acetynitrile, 100 mM fumic acid pH 3.0, and analyzed by nano LC-MS/MS system for sequencing. A high-pressure liquid chromoatography C18 column was coupled with an ion-trap mass spectometer. The MS/MS data were analyzed with Protech's proprietary software. Peptide containing IRF5 serine 309 was identified by LS/MS/MS to be phosphorylated in the presence of TBK-1 *in vivo*. Additional *in vivo* phosphorylation analyses were performed by co-transfection of T7-His-IRF5 with either myc-TBK-1, myc-TRAF6, or HA-RIP2 in HEK293 cells. IRF5 was collected on T7 antibody agarose beads from the individual transfections, eluted, and subsequently pooled. Proteins were separated by SDS-PAGE, visualized with Commassie brilliant blue stain, treated with iodoacetamide, and submitted for analysis to ProtTech Inc. as described. Peptide analyses by LS/MS/MS identified additional phosphorylated amino acids corresponded to threonine 10, serine 309, serine 451, and serine 462. The detectable b-type and y-type fragment ions for the phosphopeptides were annotated in Figure S2, and bold print indicates key fragments to facilitate identification of phosphorylation residues.

## Supporting Information

Figure S1 **Transfection controls for protein expression in** **Figure 1a** **.** Cell lysates from a transfection shown Figure 1a indicate similar levels of IRF5 are expressed with co-expression of c-myc-tagged TBK-1, c-myc-tagged TRAF6 or omini-tagged RIP2. Western blots with anti-IRF5, anti-myc or anti-omni antibodies.
(TIF)

Figure S2 **IRF5 phosphorylated amino acids identified by LC-MS/MS.** Phosphopeptides identified are shown with specific phosphorylated threonine or serine. **a–e**) Identification of *in vivo* phosphorylated amino acids, threonine 10, serine 309, serine 317, serine 451, and serine 462 by ProtTech, Inc. following IRF5 immunoprecipitation from HEK293 cells expressing TBK1, TRAF6, and RIP. (see Methods). **f**) Identification of serine 158 phosphorylation by *in vitro* kinase reaction with TBK-1. Analysis by University of Massachusetts Proteomics Lab.
(TIF)

Figure S3 **Transfection controls for IRF5 expression in** **Figure 3** **.** Expression levels of wt IRF5 and IRF5 mutations in absence or presence of omini-tagged RIP2 are similar in luciferase reporter studies shown in Figure 3b (**a**) and 3d (**b**). Western blots with anti-IRF5, anti-omni or anti-tubulin antibodies.
(TIF)

Figure S4 **Polyubiquitination of IRF5 via ubiquitin lysine 63.** Assay performed as described in Fig. 6b. With co-expression of HA-tagged ubiquitin K0R63K (K63Ub).
(TIF)

Figure S5 **Expression controls for IRF5 in** **Figure 7** **.** Expression levels are similar for wt IRF5, IRF5 KK427,428RR (KK/RR), and IRF5 KK427,428RR, SS451,462DD (KK/RR, SS/DD). Proteins from an experiment of Figure 7 were evaluated by Western blot with indicated antibodies.
(TIF)

Figure S6 **Controls for IRF5 expression in apoptosis studies of** **Figure 8** **.** Western blot of cell lysates from experiment shown in Figure 8c with anti-IRF5 or anti-tubulin antibodies.
(TIF)

Table S1 **Oligonucleotide primers for plasmid generation.** Single stranded forward primer DNA sequences are shown (5′ to 3′) that were used to introduce mutations in the IRF5 DNA sequence.
(PDF)
