# Supplementary figures and images for: Activation of Interferon Regulatory Factor 5 by Site Specific Phosphorylation
Source: PLoS One. 2012 Mar 8;7(3):e33098. doi: 10.1371/journal.pone.0033098 (PMC3297630; doi:10.1371/journal.pone.0033098)

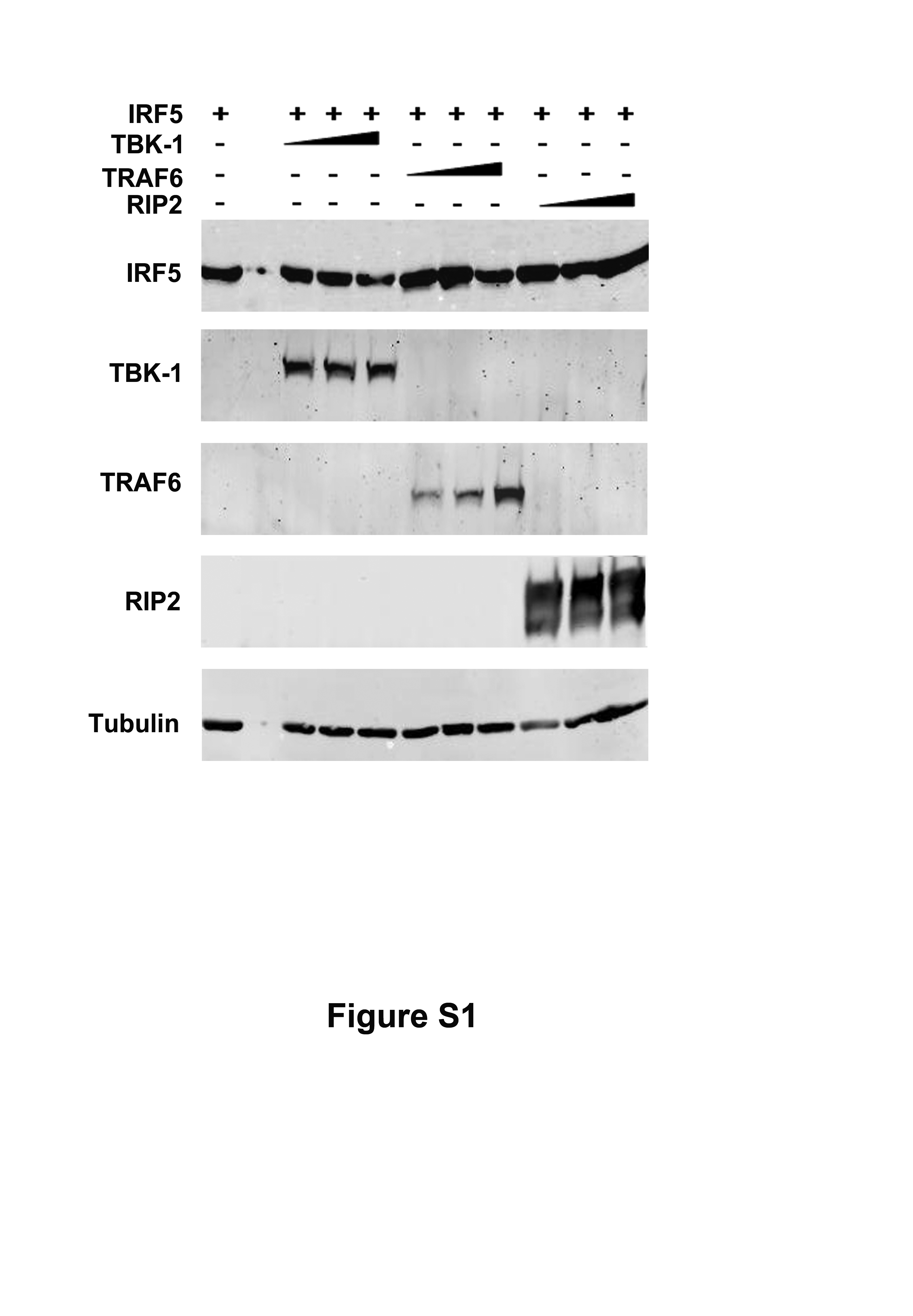

Supplement: Figure S1 — Transfection controls for protein expression in Figure 1a . Cell lysates from a transfection shown Figure 1a indicate similar levels of IRF5 are expressed with co-expression of c-myc-tagged TBK-1, c-myc-tagged TRAF6 or omini-tagged RIP2. Western blots with anti-IRF5, anti-myc or anti-omni antibodies. (TIF) [file pone.0033098.s001.tif]

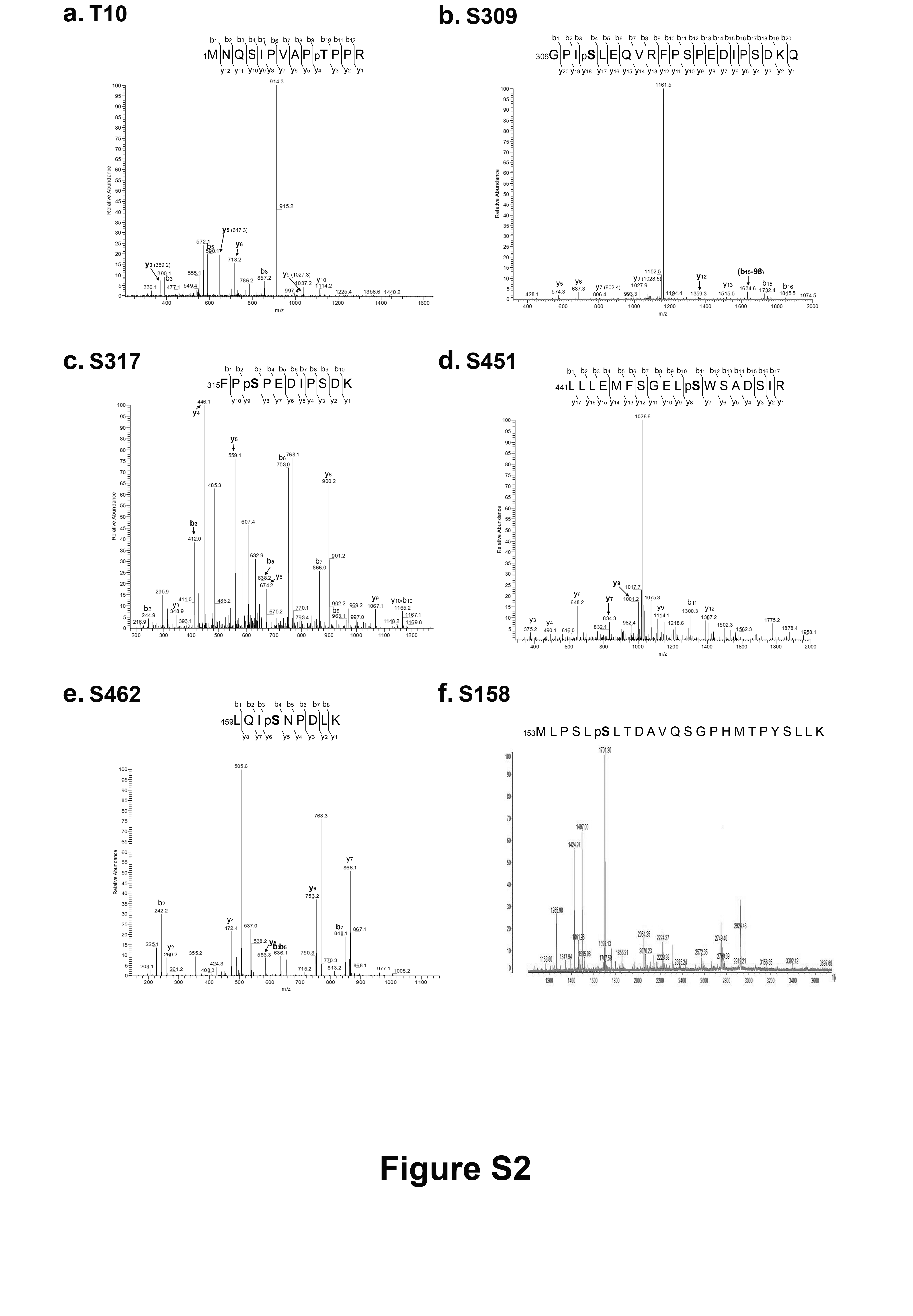

Supplement: Figure S2 — IRF5 phosphorylated amino acids identified by LC-MS/MS. Phosphopeptides identified are shown with specific phosphorylated threonine or serine. a–e) Identification of in vivo phosphorylated amino acids, threonine 10, serine 309, serine 317, serine 451, and serine 462 by ProtTech, Inc. following IRF5 immunoprecipitation from HEK293 cells expressing TBK1, TRAF6, and RIP. (see Methods). f) Identification of serine 158 phosphorylation by in vitro kinase reaction with TBK-1. Analysis by University of Massachusetts Proteomics Lab. (TIF) [file pone.0033098.s002.tif]

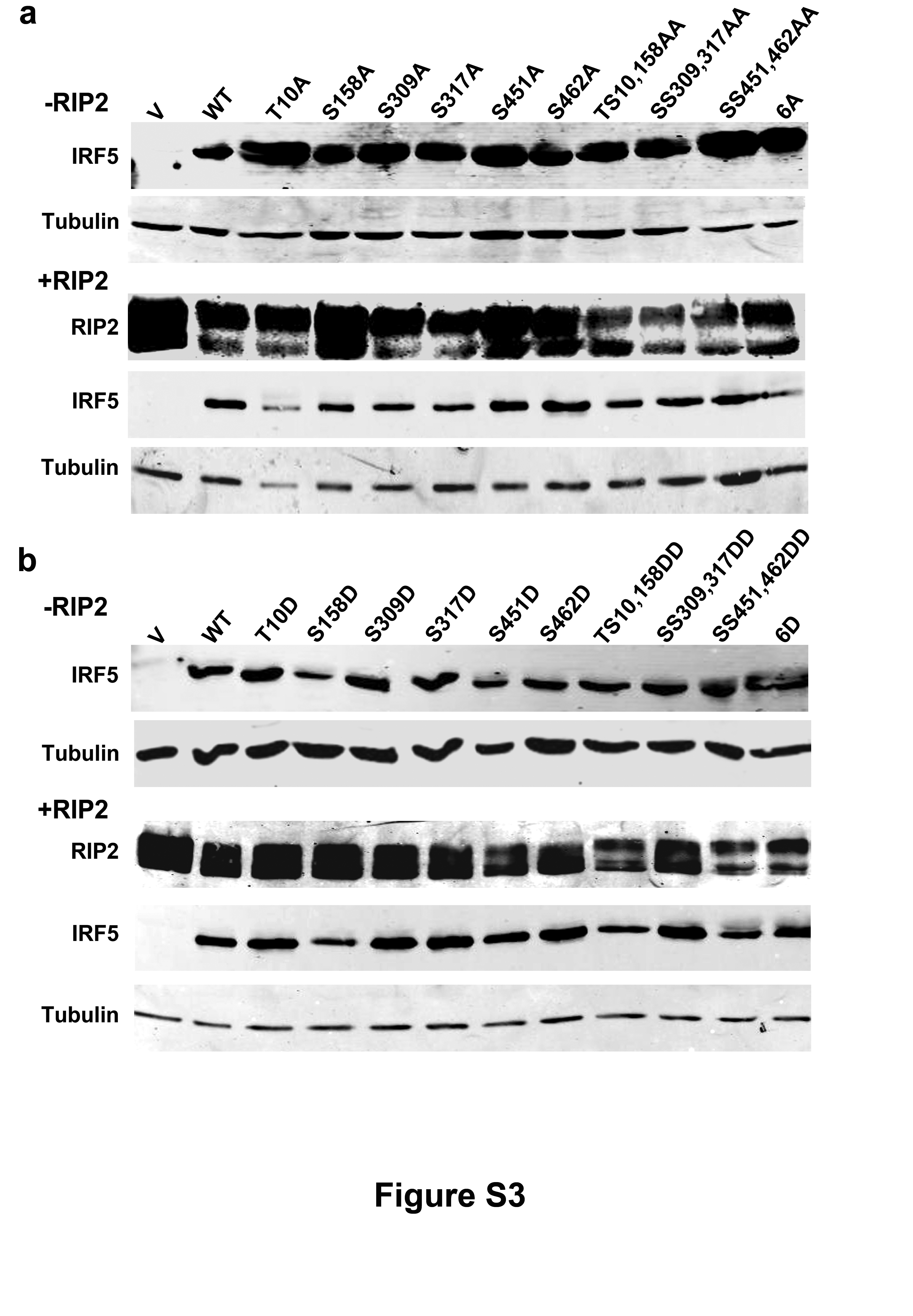

Supplement: Figure S3 — Transfection controls for IRF5 expression in Figure 3 . Expression levels of wt IRF5 and IRF5 mutations in absence or presence of omini-tagged RIP2 are similar in luciferase reporter studies shown in Figure 3b (a) and 3d (b). Western blots with anti-IRF5, anti-omni or anti-tubulin antibodies. (TIF) [file pone.0033098.s003.tif]

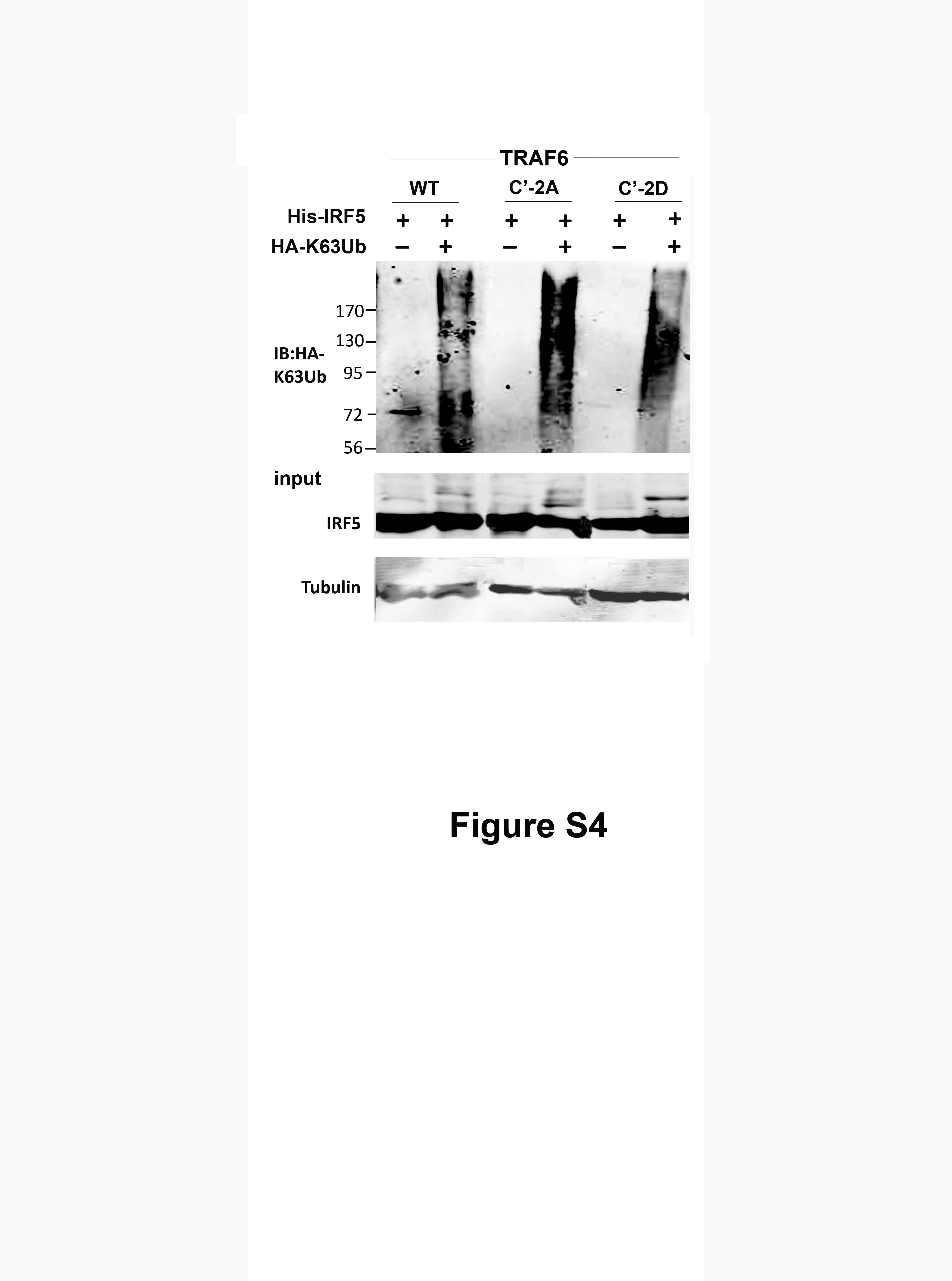

Supplement: Figure S4 — Polyubiquitination of IRF5 via ubiquitin lysine 63. Assay performed as described in Fig. 6b. With co-expression of HA-tagged ubiquitin K0R63K (K63Ub). (TIF) [file pone.0033098.s004.tif]

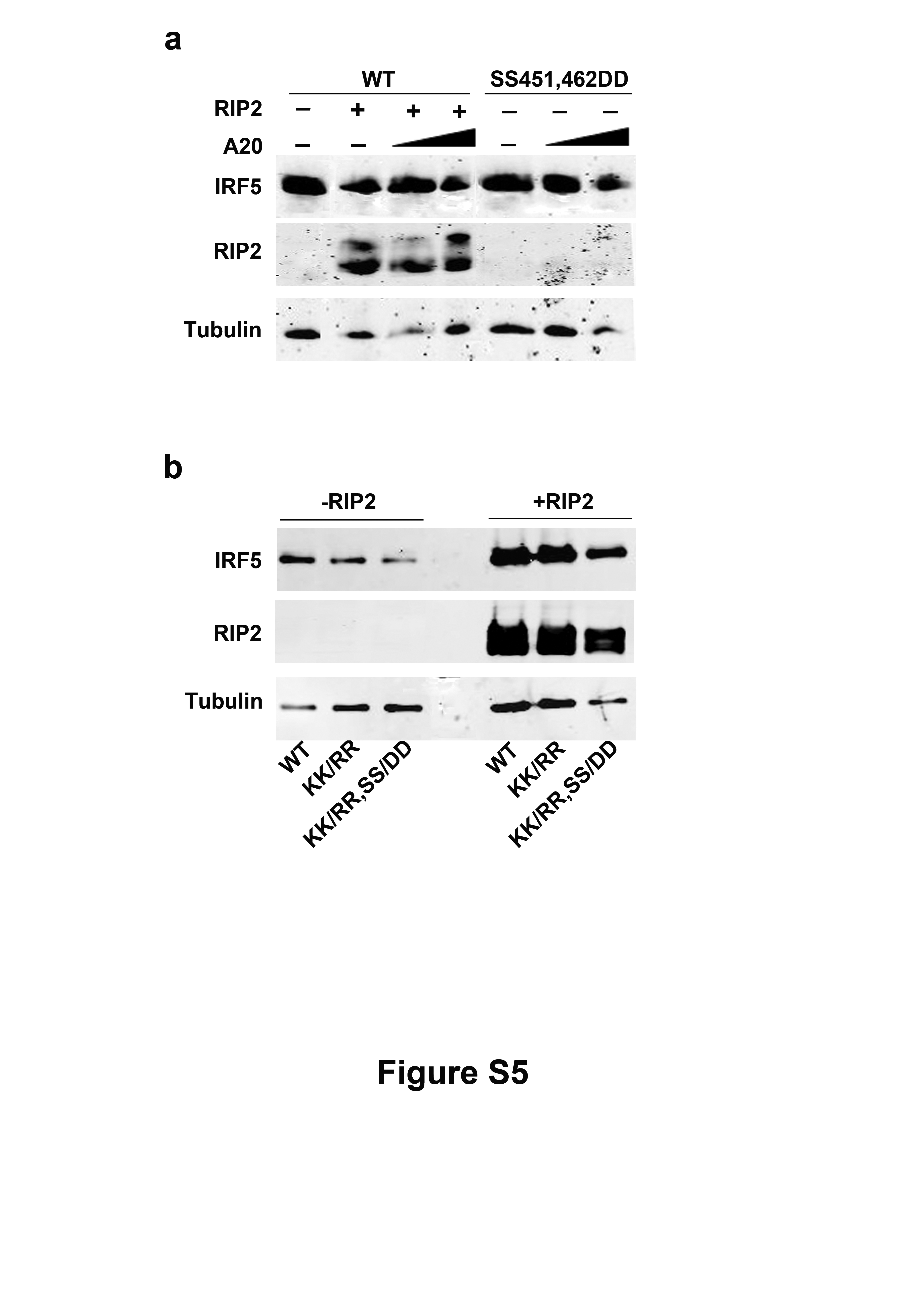

Supplement: Figure S5 — Expression controls for IRF5 in Figure 7 . Expression levels are similar for wt IRF5, IRF5 KK427,428RR (KK/RR), and IRF5 KK427,428RR, SS451,462DD (KK/RR, SS/DD). Proteins from an experiment of Figure 7 were evaluated by Western blot with indicated antibodies. (TIF) [file pone.0033098.s005.tif]

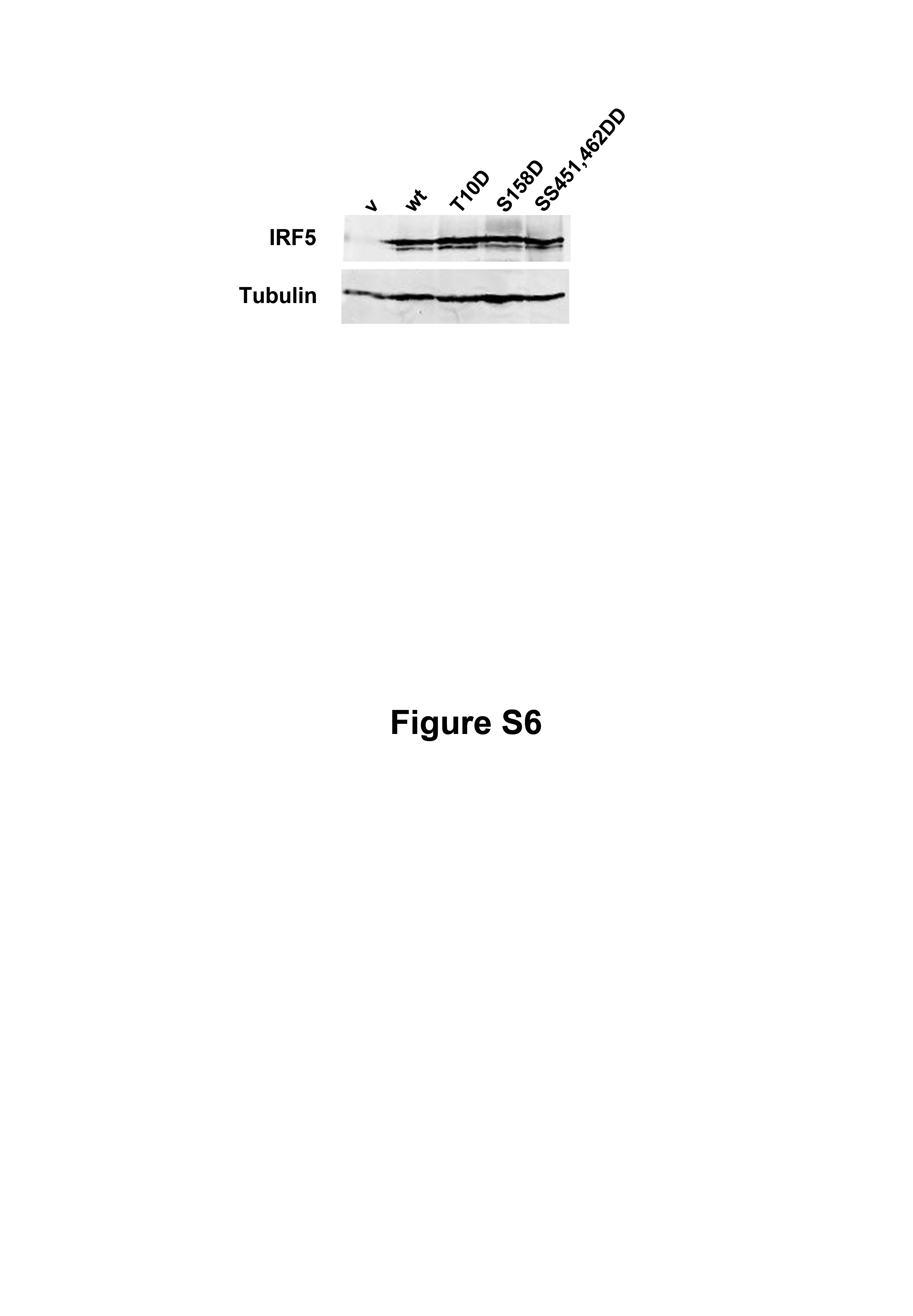

Supplement: Figure S6 — Controls for IRF5 expression in apoptosis studies of Figure 8 . Western blot of cell lysates from experiment shown in Figure 8c with anti-IRF5 or anti-tubulin antibodies. (TIF) [file pone.0033098.s006.tif]
