# Supplementary material for: Activation of Interferon Regulatory Factor 5 by Site Specific Phosphorylation
Source: PLoS One. 2012 Mar 8;7(3):e33098. doi: 10.1371/journal.pone.0033098 (PMC3297630; doi:10.1371/journal.pone.0033098)
Supplement: Table S1 — Oligonucleotide primers for plasmid generation. Single stranded forward primer DNA sequences are shown (5′ to 3′) that were used to introduce mutations in the IRF5 DNA sequence. (PDF) [file pone.0033098.s007.pdf]

Supplement Table 1

Primers used for the site-directed mutagenesis and the DNA cloning.
(Forward primers are shown)

#	Name	Primers 5' – 3'	Purpose
1	F IRF5v5T10A	CCA GTG GCT CCC GCC CCA CCC CGC CGC	T10A
2	F IRF5v5 S158A	TTG CCA AGC CTG GCC CTC ACA GAT GCA	S158A
3	F IRF5v5 S309A	TTC GGC CCC ATA GCC CTG GAG CAA GTG	S309A
4.	F IRF5v5 S317A	GTG CGC TTC CCC GCC CCT GAG GAC ATC	S317A
5.	F IRF5v5 S451A	TCA GGG GAG CTA GCT TGG TCA GCT GAT	S451A
6.	F IRF5v5 S462A	CGG CTA CAG ATC GCA AAC CCA GAC CTC	S462A
7.	F IRF5v5T10D	CCA GTG GCT CCC GAC CCA CCC CGC CGC	T10D
8.	F IRF5v5 S158D	TTG CCA AGC CTG GAC CTC ACA GAT GCA	S158D
9.	F IRF5v5 S309D	TTC GGC CCC ATA GAC CTG GAG CAA GTG	S309D
10.	F IRF5v5 S317D	GTG CGC TTC CCC GAC CCT GAG GAC ATC	S317D
11.	F IRF5v5 S451D	TCA GGG GAG CTA GAC TGG TCA GCT GAT	S451D
12.	F IRF5v5 S462D	CGG CTA CAG ATC GAC AAC CCA GAC CTC	S462D
13.	F IRF5v5 KK/RR	CGC AAA CCC CGA GAG CGG CGG CTC ATT ACT GTA	K K 427,428RR
14.	ΔN IRF5 -1	ATC GCT TAA GTG CTC CAA TGG CCC TGC TC	ΔN IRF5
15.	ΔN IRF5 -2	ATC TAG ATT GCA TGC CAG CTG GGT A	ΔN IRF5
